# Low-intensity pulsed ultrasound ameliorates glia-mediated inflammation and neuronal damage in experimental intracerebral hemorrhage conditions

**DOI:** 10.1186/s12967-023-04377-z

**Published:** 2023-08-24

**Authors:** Wei-Shen Su, Chun-Hu Wu, Wen-Shin Song, Szu-Fu Chen, Feng-Yi Yang

**Affiliations:** 1 Department of Biomedical Imaging and Radiological Sciences, School of Biomedical Science and Engineering, National Yang Ming Chiao Tung University, No. 155, Sec. 2, Li-Nong Street, Taipei, 11221 Taiwan; 2 Institute of Biomedical Sciences, Academia Sinica, Taipei, Taiwan; 3 Division of Neurosurgery, Cheng Hsin General Hospital, Taipei, Taiwan; 4 Department of Physical Medicine and Rehabilitation, Cheng Hsin General Hospital, No. 45, Cheng Hsin Street, Taipei, 11221 Taiwan; 5 Department of Physiology and Biophysics, National Defense Medical Center, Taipei, Taiwan; 6 Department of Neurological Surgery, Tri-Service General Hospital, National Defense Medical Center, Taipei, Taiwan

**Keywords:** Intracerebral hemorrhage, Low-intensity pulsed ultrasound, Neuroprotective effects, Neuron inflammation

## Abstract

**Background:**

Intracerebral hemorrhage (ICH) is a condition associated with high morbidity and mortality, and glia-mediated inflammation is a major contributor to neurological deficits. However, there is currently no proven effective treatment for clinical ICH. Recently, low-intensity pulsed ultrasound (LIPUS), a non-invasive method, has shown potential for neuroprotection in neurodegenerative diseases. This study aimed to investigate the neuroprotective effects and potential mechanisms of LIPUS on glia-mediated inflammation in ICH.

**Methods:**

This study used 289 mice to investigate the effects of LIPUS on ICH. ICH was induced by injecting bacterial collagenase (type VII-S; 0.0375 U) into the striatum of the mice. LIPUS was applied noninvasively for 3 days, including a 2-h-delayed intervention to mimic clinical usage. The study evaluated neurological function, histology, brain water content, hemoglobin content, MRI, and protein expression of neurotrophic factors, inflammatory molecules, and apoptosis. In vitro studies investigated glia-mediated inflammation by adding thrombin (10 U/mL) or conditioned media to primary and cell line cultures. The PI3K inhibitor LY294002 was used to confirm the effects of PI3K/Akt signaling after LIPUS treatment.

**Results:**

LIPUS treatment improved neurological deficits and reduced tissue loss, edema, and neurodegeneration after ICH. The protective effects of LIPUS resulted from decreased glia-mediated inflammation by inhibiting PI3K/Akt-NF-κB signaling, which reduced cytokine expression and attenuated microglial activation-induced neuronal damage in vitro.

**Conclusions:**

LIPUS treatment improved neurological outcomes and reduced glia-mediated inflammation by inhibiting PI3K/Akt-NF-κB signaling after ICH. LIPUS may provide a non-invasive potential management strategy for ICH.

**Supplementary Information:**

The online version contains supplementary material available at 10.1186/s12967-023-04377-z.

## Background

Intracerebral hemorrhage (ICH) is a devastating sub-type of cerebral stroke with high mortality and morbidity [1] that eventually causes brain edema, tissue damage, and neurological deficits [2]. No treatment has yet been proven effective for clinical ICH [2]. Thus, preventing ICH damage remains an urgent issue worldwide. Low brain-derived neurotrophic factor (BDNF) and high vascular endothelial growth factor (VEGF) levels in serum are risk factors for cerebroventricular disease [3]. BDNF was downregulated in experimental ICH [4] and VEGF was upregulated in clinical ICH patients [5]. In pathological ICH processes, the activation of the inflammatory response after vessel disruption-induced injury crucially contributes to neuronal death after ICH [6]. Microglia are the major immune cells in the central nervous system (CNS) after an inflammatory response [6, 7]. Inhibiting microglial activation has been reported to reduce cell death, tissue damage, and motor and neurological deficits [8]. Thrombin is one of the important factors produced through a coagulation cascade to trigger cytotoxic effects, oxidative stress, and inflammation [1]. Thrombin-induced inflammatory effects are primarily mediated by protease-activated receptors (PARs) [9]. Our previous study found that microglia exert neurotoxic effects after thrombin induction [10]. Unlike the protective effects PI3K/Akt signaling exerts on neurons, emerging evidence reports that the PI3K/Akt pathway contributes to microglia-induced neuroinflammation [11]. Upregulated Akt phosphorylation is related to increased inflammatory factors of interleukin (IL)-1β, IL-6, and inducible nitric oxide synthase (iNOS) expression after microglial activation [12]. Mechanistically, The NF-κB signaling pathway plays a major role in microglial activation to express cytokines such as IL-6 and macrophage inflammatory protein (MIP)-2 [10]. Whether microglial activation after thrombin induction occurs through PI3K/Akt phosphorylation and NF-κB activation must be investigated. Previous studies found that treatment with thrombin and PAR-4 AP could induce NF-κB activation in N9 microglial cells [9]. Activating the PI3K/Akt signaling cascade can trigger NF-κB signaling pathway upregulation in BV2 microglia and result in PC12 neuronal death [13]. Moreover, inhibiting PI3K/Akt signaling with LY294002, a specific PI3K/Akt inhibitor, reduced microglia-caused cytokine expression [12]. In addition to microglia, astrogliosis also participates in the pathophysiology of ICH [14]. However, the role of astrocyte in ICH remains unknown. Astrocytes could improve motor outcomes by overexpressing heme oxygenase-1 after ICH [15]; on the other hand, suppressing astrocyte activity alleviated the neurobehavioral deficit in ICH [16]. A recent study highlights the importance of astrocytes to inflammatory response-caused neurotoxic effects [17]. Thrombin could directly induce primary astrocyte activation and promote IL-6 expression [18]. Additionally, microglial activation contributes to inducing astrocyte activation. Conditioned media produced by activated microglia were able to induce astrocytic cytokine expression and triggered reactive astrocyte expression that further aggravated the neurotoxic effects [19].

Recently, low-intensity pulsed ultrasound (LIPUS), a non-invasive method with neuroprotective potential, has been suggested for many CNS-related disorders such as Alzheimer’s disease (AD) [20], Parkinson’s disease (PD) [21], and traumatic brain injury (TBI) [22, 23]. LIPUS intervention reduced AD- or TBI-induced neuronal death and TBI-induced behavioral outcomes [22, 23]. A relationship between LIPUS and the inflammatory response was found. LIPUS caused a decrease in cytokines such as IL-1β and IL-6 and NF-κB expression after debris-induced RAW 264.7 macrophage cell activation [24]. In CNS, LIPUS treatment inhibited TBI-induced microglial activation [23]. LIPUS was also able to prevent microglial and astrocyte activation in the brain cortex after LPS-caused brain injury [25, 26]. However, whether treatment with LIPUS would ameliorate ICH-induced detrimental outcomes and inflammatory responses remains to be seen. This study hypothesizes that LIPUS treatment improves ICH-induced inflammation that causes neuronal death and tissue damage. LIPUS may offer protection in ICH cases by limiting the PI3K/Akt-NF-κB signaling pathway after microglial activation and thus inhibiting the astrocyte reaction and neuronal death.

## Methods

### Animals and surgical procedures

All animal experiments were conducted according to the guidelines of and approved by the Animal Care and Use Committee of National Yang Ming Chiao Tung University. The animals were blindly randomized to different treatment groups with computer-generated random numbers. All outcome measurements described below were also performed blinded. Animals were housed at 22–25 °C and 40–60% humidity on a 12-h/12-h dark cycle and were allowed free access to water and food. Male C57BL/6 mice (9–10 weeks old, 22–28 g in weight) were intraperitoneally anesthetized with sodium pentobarbital (65 mg/kg; Rhone Merieux, Harlow, UK) and placed in a stereotaxic frame. ICH was induced with an injection of collagenase (0.0375 U of type VII-S in 1 μL of normal saline; Sigma-Aldrich, St. Louis, MO, USA) into the striatum using the stereotactic coordinates 0.8 mm anterior and 2.5 mm lateral to the bregma, 2.5 mm in depth, and at a rate of 0.1 μL/min over 10 min [10]. The needle was left in place for an additional 20 min to prevent reflux. Control mice were injected with an equal volume of saline in the same manner. Mice were placed in a heated cage to maintain body temperature while recovering from anesthesia. After the ICH or sham surgery, animals were housed under the conditions described previously.

### Experimental design

**Fig. 1:**
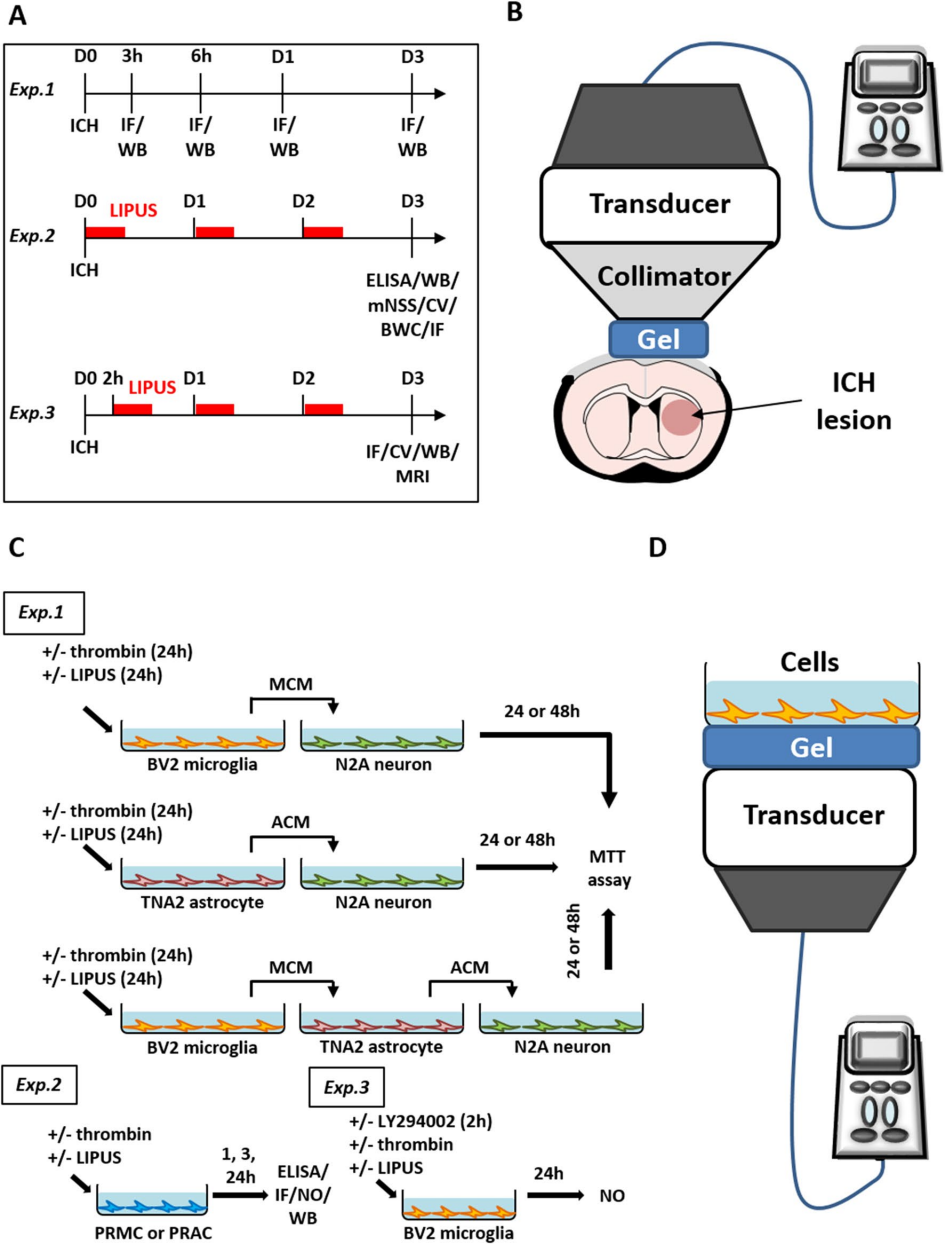
Experimental design & ultrasound apparatus.** A** Experimental design of ICH animal model: Exp.1) Expressions of Iba1 (microglia) and GFAP (astrocyte) were measured at 3 h, 6 h, 1 day, and 3 days after ICH; Exp.2) LIPUS treatment (red dash) was performed daily from D0 to D2 in the brain, and mice were sacrificed on D3; Exp.3) 2-h-delayed LIPUS treatment post-ICH. **B** Schematic diagram of LIPUS for ICH animal model. The ultrasound transducer was coupled with a metal collimator to target on the ICH lesion. **C** Experimental design of cell cultures: Exp.1) The study involved the following cell types: BV2 (microglia), TNA2 (astrocyte), and N2A (neuron). BV2 cells were stimulated with thrombin (10 U/mL), TNA2 cells were stimulated with either thrombin (10 U/mL) or microglia-conditioned media (MCM), and N2A cells were stimulated with either MCM or astrocyte-conditioned media (ACM). After the induction, cells received LIPUS treatment, and cell viability was measured at 24 h or 48 h. Additionally, conditioned media were collected for 24 h following the induction. Exp.2) Primary cell cultures were subjected to thrombin stimulation (10 U/mL) and either treated or not treated with LIPUS. The cells were then harvested at 1 h, 3 h, or 24 h following the stimulation. Exp.3) The LY294002 (PI3K inhibitor) was administered 2 h prior to inducing thrombin stimulation (10 U/mL) with or without LIPUS treatment. The cells were then harvested after 24 h. **D** Schematic diagram of LIPUS treatment of cell cultures. Acoustic wave was sonicated from the bottom of culture plate to stimulate cells. (*IF* immunofluorescence staining, *WB* western blots, *CV* cresyl violet staining, *mNSS* modified neurological severity score, *BWC* brain water content, *NO* nitrite oxide, *MTT* 3-(4,5-dimethyl-2-thiazolyl)- 2,5-diphenyl-2H-tetrazolium bromide assay)

A total of 289 mice were used in this study, divided into 3 groups: (Fig. 1A). Three experiments were conducted. (Experiment 1) The time-course expression of microglia and astrocytes were measured at 3 h, 6 h, 1 day, and 3 days after ICH by western blot and immunostaining (n = 5/group). (Experiment 2) Levels of cytokines measured by ELISA (n = 7–9/group), neurotrophic factors and death markers measured by western blotting (n = 7–8/group), modified neurological severity score (mNSS) (n = 8/group), injury volumes measured by cresyl violet staining (n = 8/group), brain edema measured by brain water content (n = 8/group), degenerating neurons measured by FJB staining (n = 8/group) and myelin, microglia, and astrocytes measured by immunostaining (n = 8/group) were assessed 3 days after treatment with LIPUS post-ICH. (Experiment 3) Levels of injury volume measured by cresyl violet staining (n = 7/group), brain lesion volume measured by MRI (n = 7/group), degenerating neurons measured by FJB staining (n = 7/group), and myelin, microglia, and astrocytes measured by immunostaining (n = 7/group) were assessed 3 days after 2-h-delayed LIPUS treatment post-ICH.

### Pulsed ultrasound apparatus

LIPUS sonication was generated with a therapeutic ultrasound apparatus (ME740, Mettler Electronics, CA, USA) with a 1.0-MHz. In vivo study, single-element transducer was coupled with a cone-shaped metal ultrasound collimator (Fig. 1B). Ultrasound coupling gel was smeared on the interface between the transducer and the mouse. The mice were anesthetized with 1%–2% isoflurane during the sonication procedure. A stereotaxic apparatus (Stoelting, Wood Dale, IL, USA) and ultrasound collimator precisely targeted the transducer on the needle entry location to cover all of the injured areas. LIPUS treatment was administered either as a single 5-min sonication session or as three 5-min sonication sessions with two 5-min rest intervals in between. The transducer was applied with a 2-ms burst length, a duty cycle of 20%, and a repetition frequency of 100 Hz ± 5%. The spatial-peak temporal-average intensity (${I}_{SPTA}$) used for the treatment was 528mW/cm^2^. The intensity of the LIPUS was selected based on data from our previous studies [22, 23] and there was no significant tissue damage or inflammatory response in normal mice [22].

### BV2, CTX TNA2, and neuro-2A (N2A) cell line cultures

As in the previous studies [10, 27], the BV2 microglial, CTX TNA2 astrocyte, and N2A neuronal cell lines were cultured in DMEM (Gibco) supplemented with 10% heat-inactivated FBS (Gibco), 50 U/mL penicillin, and 50 μg/mL streptomycin in a humidified atmosphere of 5% CO_2_ at 37 °C.

### Primary microglia culture

As in our previous study [28], the primary rat microglial culture was obtained from P7 post-natal rat brain striatum and cultured in Dulbecco’s Modified Eagle medium (DMEM; Gibco, Bethesda, MD, USA) supplemented with 10% heat-inactivated fetal bovine serum (FBS; Gibco), 50 U/mL penicillin, and 50 μg/mL streptomycin in a humidified atmosphere of 5% CO_2_ at 37 °C. Microglial cells were ready for use after 14 days of culture.

### Primary astrocyte culture

The primary rat astrocyte culture was obtained from P7 post-natal rat brain striatum as previously described [29] and cultured in DMEM (Gibco) supplemented with 10% heat-inactivated FBS (Gibco), 50 U/mL penicillin, and 50 μg/mL streptomycin in a humidified atmosphere of 5% CO_2_ at 37 °C. After 14 days of culture, the media were collected and transferred to 6 cm dishes for further culture; the astrocyte cells were ready for use after an additional 14 days of culture.

### Culture drug and LIPUS treatment

In order to investigate the potential protective mechanism of LIPUS in ICH, thrombin was added or conditioned media transferred to induce glia-mediated inflammation in primary and cell line cultures (Fig. 1C). (Experiment 1) BV2 cells were stimulated with thrombin (10 U/mL); TNA2 cells were stimulated with thrombin (10 U/mL) or microglia-conditioned media (MCM), and N2A cells were stimulated with MCM or astrocyte-conditioned media (ACM). After induction, cells received 15-min LIPUS treatment and were measured at 24 h or 48 h. Conditioned media were collected after induction for 24 h. (Experiment 2) Primary cultures were stimulated with thrombin (10 U/mL) in the absence or presence of LIPUS treatment and harvested at 1 h, 3 h, or 24 h. (Experiment 3) LY294002 (50 μM, Cell Signaling, Danvers, MA, USA) was treated 2 h before thrombin (10 U/mL) induction in the absence or presence of LIPUS treatment and harvested at 24 h. The experiments were repeated three to five times with independent cultures. During the in vitro study, LIPUS was applied by sonication from the bottom of the culture plate to stimulate the cells (Fig. 1D). To ensure optimal ultrasound transmission, an ultrasound coupling gel was applied between the transducer and the culture plate. Our previous study showed that LIPUS at a ${I}_{SPTA}$ of 30mW/cm^2^ would not impact the adhesion and cell proliferation of microglia [26].

### Modified neurological severity score

The mNSS, as in our previous study [30] with minor modification, included sensory, reflex, motor, and balance tests and was performed before ICH and at 1 day and 3 days post-ICH. Neurological function was scored on a scale of 0–17 (normal score: 0).

### Brain water content

Brain edema was evaluated by measuring brain water content [4]. Brain tissues were removed immediately under anesthesia and divided into five parts, consisting of the ipsilateral and contralateral cortex, ipsilateral and contralateral basal ganglia, and the cerebellum, which served as an internal control. Tissue samples were weighed (wet weight), then baked at 100 °C for 24 h and reweighed (dry weight). Water content was determined as [(wet weight-dry weight)/wet weight] × 100%.

### Hemoglobin assay

The hemoglobin content of brains after ICH was measured through a spectrophotometric assay as in a previous study [4]. Mice were transcardially perfused and the ipsilateral striatum regions were collected after ICH. Distilled water (300 μL) was added to the collected tissues and they were homogenized for 30 s followed by sonication on ice for 1 min. After centrifugation at 13,000 rpm for 30 min, 60 μL of supernatant was mixed with Drabkin’s reagent (240 μL, Sigma-Aldrich) for 15 min at room temperature. Optical density (545 nm) was then measured to assess the concentration of cyanmethemoglobin. To generate a standard curve, blood was collected by cardiac punctures from anesthetized control mice. Incremental volumes of blood (0, 0.5, 1.0, 2.0, 4.0, and 8.0 μL) were then added to 300 μL of tissue lysate from a normal hemispheric sample.

### MRI analysis

The study utilized a 7 T PET/MR system (BioSpec AVNEO 70/18 PETMR INLINE, Bruker) to conduct MRI studies. T2-weighted images (T2WI) were obtained on days 1 and 3 post-ICH to assess lesion volumes. T2WIs were rapidly acquired with a relaxation enhancement sequence (RARE). The image parameters were: bandwidth = 35 kHz, repetition time (TR)/echo time (TE) = 2654/35 ms, number of averages = 4, matrix size = 256 × 256, field of view (FOV) = 22 × 22 mm, and section thickness = 0.5 mm. The imaging plane was positioned across the center of the lesion site. After normalizing the image intensities pre- and post-ICH, the regions of interest (ROI) were identified by the contrast provided by T2WI between lesions and brain tissues [16]. A blinded operator delineated the contours of hyperintense areas with the ImageJ software version 1.53 g (ImageJ, National Institutes of Health, Bethesda, MD, USA) ROI tool, with > 2 SDs signal intensity of contralateral normal tissue as a buffer [31, 32]. Lesion volume was assessed by summing up the injury area measured from 6 slices and multiplying by the slice thickness (0.5 mm) from T2WIs.

### Tissue processing and histology

Mice were transcardially perfused with normal saline followed by 4% paraformaldehyde after terminal anesthesia. Brains were removed, post-fixed in 4% paraformaldehyde overnight, cryoprotected with 30% sucrose, and then sectioned coronally (10 μm) from the level of the olfactory bulbs to the visual cortex.

### Injury volume and hemispheric enlargement assessment

Injury volumes and hemispheric enlargement ratios were quantified using coronal sections stained with cresyl violet at 20 rostral-caudal levels that were spaced 200 μm apart. Sections were analyzed using ImageJ software version 1.50i (ImageJ, National Institutes of Health, Bethesda, MD, USA). Volume measurement was computed by summing the areas and multiplying the total by the interslice distance (200 μm). Hemispheric atrophy was assessed as ([contralateral hemisphere or striatal volume—ipsilateral hemisphere or striatal volume]/contralateral hemisphere or striatal volume) × 100%. Hemispheric enlargement was assessed as ([ipsilateral hemisphere volume—contralateral hemisphere volume]/contralateral hemisphere volume) × 100%. Two experimenters who were blinded to all animal groups performed the analysis. The inter-rater reliability was within 10%.

### Fluoro-Jade B staining

Fluoro-Jade B (FJB; Chemicon, Temecula, CA, USA) is a polyanionic fluorescein derivative that labels degenerating neurons with high sensitivity and specificity [33]. Sections were rehydrated in graded ethanol solutions (100% and 70% for 5 min each) and distilled water, incubated in 0.06% KMnO_4_ followed by a 0.001% solution of FJB for 30 min each, and observed under a fluorescence microscope (Olympus BX-51; Olympus, Tokyo, Japan) at 450–490 nm.

### Immunofluorescence staining

(1) Immunofluorescence labeling was performed by simultaneously incubating sections with rabbit anti-ionized calcium-binding adaptor molecule 1 (Iba1; a microglia/macrophage marker; 1:1000; Wako, Richmond, VA, USA), rat anti-glial fibrillary acidic protein (GFAP; an astrocyte marker; 1:500; Invitrogen, Camarillo, CA, USA), or anti-myelin basic protein (MBP; 1:200, Abcam, Cambridge, UK) overnight at 4 °C. (2) Double immunofluorescence labeling was performed by simultaneously incubating slices with rabbit anti-PAR4 followed by mouse anti-OX-42 (1:100, Abcam) or mouse anti-C3 (1:100, Santa Cruz, Santa Cruz, CA, USA) followed by rat anti-GFAP (1:500, Invitrogen). Sections and slices were washed and then incubated with Alexa Fluor 488- or Alexa Fluor 594-conjugated secondary antibodies (1:500; Molecular Probes, Eugene, OR, USA) for 2 h and observed under a fluorescence microscope (Olympus BX-51).

### Quantification of FJB and immunostaining

The numbers of FJB- or Iba1-positive cells and levels of GFAP or MBP intensity were quantified on three consecutive sections from the hemorrhagic core at a bregma level of 0.24 mm for each mouse. A field of 920 × 690 μm^2^ was calculated immediately adjacent to the hematoma at 200 × magnification, and non-overlapping field images around the hemorrhage clot were captured in three views as Additional File 1: Figure S1 described. The total number of FJB-positive cells and Iba1-positive cells and the mean intensity of GFAP and MBP were expressed per field of view. Two experimenters who were blinded to all animal groups performed the analysis. The inter-rater reliability was within 10%.

### Western blotting

Western blot analysis was performed as previously described [34]. A 3–5-mm coronal section from the injured hemisphere was collected after ICH or sham surgery. Cell cultures were collected at 1 h, 3 h, or 24 h after thrombin-induced activation. All samples were centrifuged at 14,000 g for 30 min and the supernatants were used for further protein analysis. The protein concentration was determined with Bradford reagent at 595 nm. Protein samples were denatured in gel-loading buffer at 100 °C for 5 min, separated by electrophoresis on sodium dodecyl sulfate–polyacrylamide gels, and transferred to Immobilon-P membranes (Millipore). Membranes were blocked with 5% milk in PBS-XT and probed overnight at 4 °C with primary antibodies including rabbit anti-cyclooxygenase-2 (COX-2, 1:1000, Cayman, Ann Arbor, MI, USA), anti-P65 (1:1000, Santa Cruz), rabbit anti-cleaved caspase-3 (cCP-3, 1:1000), rabbit anti-p-P65(1:1000), rabbit anti-pAkt Ser473 (1:1000), rabbit anti-pAkt Thr308 (1:1000), and rabbit anti-total Akt (1:1000) from Cell Signaling; rabbit anti-Iba1 (1:1000; Wako), rat anti-GFAP (1:500; Invitrogen), rabbit anti-BDNF (1:1000), and rabbit anti-VEGF (1:1000) from Genetex (Irvine, CA, USA); and mouse anti-β-actin (1: 10,000, Sigma-Aldrich). Protein band intensities were quantified with ImageJ software and were normalized to the corresponding β-actin intensity.

### ELISA

A 3–5-mm coronal section was taken from the injured hemisphere or sham animals post-ICH. IL-1β, IL-6, or MIP-2 was measured in brain homogenates with a commercially available enzyme-linked immunosorbent assay (ELISA) kit (R&D Systems, Minneapolis, MN, USA).

### NO production and cell viability

Nitrite oxide (NO) production was evaluated by measuring the nitrite levels of the culture supernatants using the Griess reagent (Sigma-Aldrich). The nitrite content in the samples was calculated based on a standard curve prepared with known concentrations of sodium nitrite. Cell viability was measured with a 3-(4,5-dimethyl-2-thiazolyl)- 2,5-diphenyl-2H-tetrazolium bromide (MTT) assay (Sigma-Aldrich). Data are presented as percentages of the control group. The experiments were repeated three to five times with cell line cultures.

### Statistical analysis

Values were expressed as mean values ± standard deviation. A Student’s t-test was used to evaluate the difference between two groups. A one-way or two-way analysis of variance (ANOVA) were used to determine significant differences among multiple groups. Bonferroni t-test was used for post hoc pairwise comparisons. The null hypothesis was rejected at *P* < 0.05.

## Results

### Microglia and astrocytes were activated after ICH

**Fig. 2:**
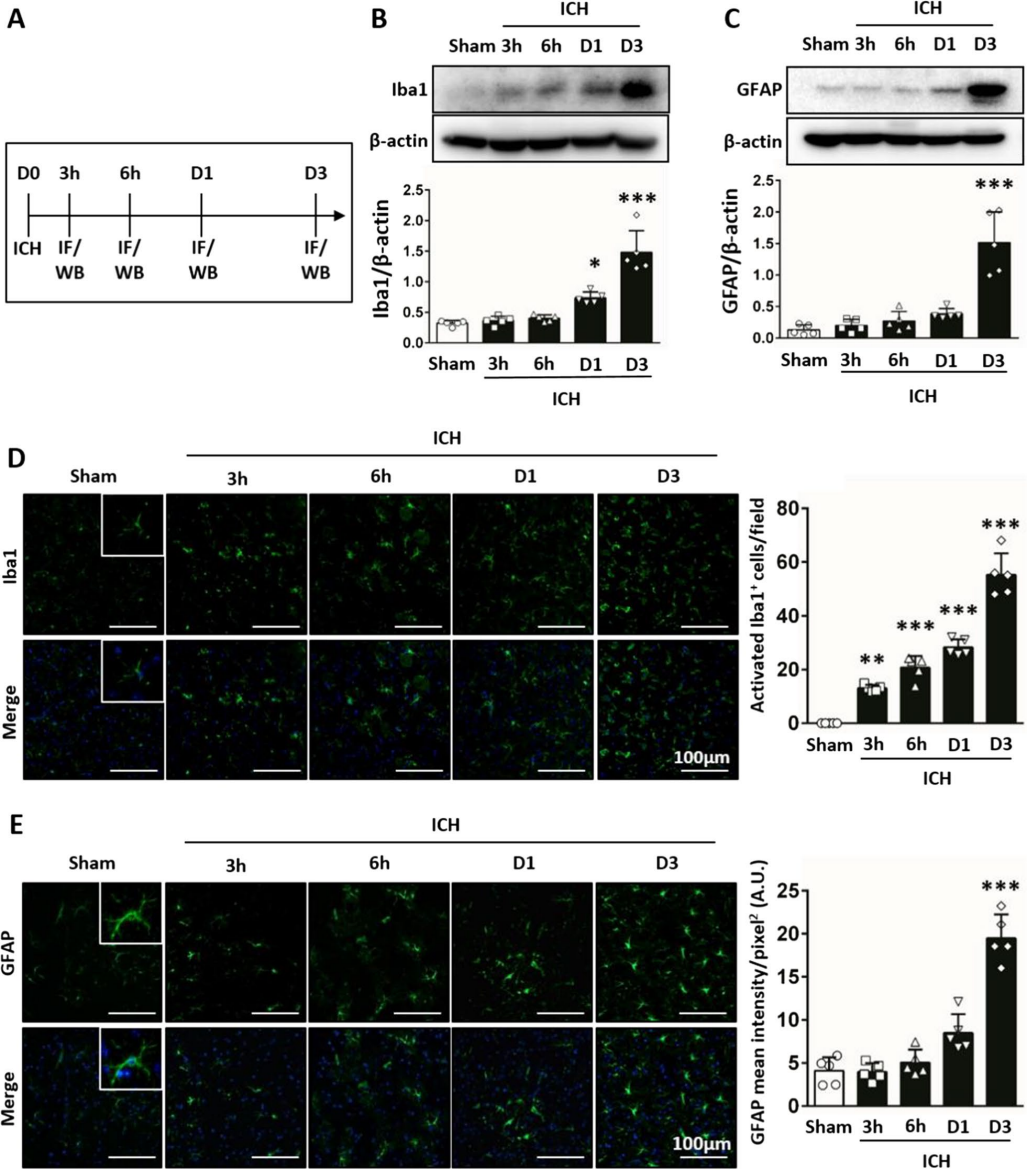
Time-course expressions of microglia and astrocyte were measured after ICH. **A** Time schedule of the study. Western blot analysis of (**B**) Iba1, and (**C**) GFAP protein levels were significantly upregulated at 1 day and/or 3 days post-ICH. Immunofluorescence double labeling for nuclei (blue) and microglia (Iba1, green) or astrocyte (GFAP, green) showed that (**D**) numbers of Iba1-positive cells activated from 3 h to 3 days, and (**E**) intensity levels of GFAP induced at 3 days post-ICH. *denotes significant difference from the sham group (*P < 0.05, **P < 0.01, ***P < 0.001; n = 5)

Microglia [10, 33] and astrocytes [16] are important factors of the inflammatory response. We first investigated the microglia and astrocyte response at different time-points after ICH. The results showed that protein levels of Iba1 were significantly upregulated from 1 to 3 days (1.48 ± 0.35 vs 0.32 ± 0.04; *P* < 0.001; Fig. 2A, B) and GFAP protein levels significantly increased at 3 days post-ICH compared with the sham group (1.51 ± 0.49 vs 0.13 ± 0.08; *P* < 0.001; Fig. 2A, C). In addition, the number of Iba1-positive cells activated increased from 3 h to 3 days (55.20 ± 8.04 vs 0.00 ± 0.00; *P* < 0.001; Fig. 2D) and the intensity levels of GFAP were induced at 3 days (19.49 ± 2.76 vs 4.09 ± 1.56; *P* < 0.001; Fig. 2E) around hematoma after ICH compared with the sham group. These data suggest that both microglia and astrocytes were activated after ICH. Therefore, the endpoint of the following experiments was placed at 3 days post-ICH.

### Treatment with LIPUS reduced inflammatory factor expression after ICH

**Fig. 3:**
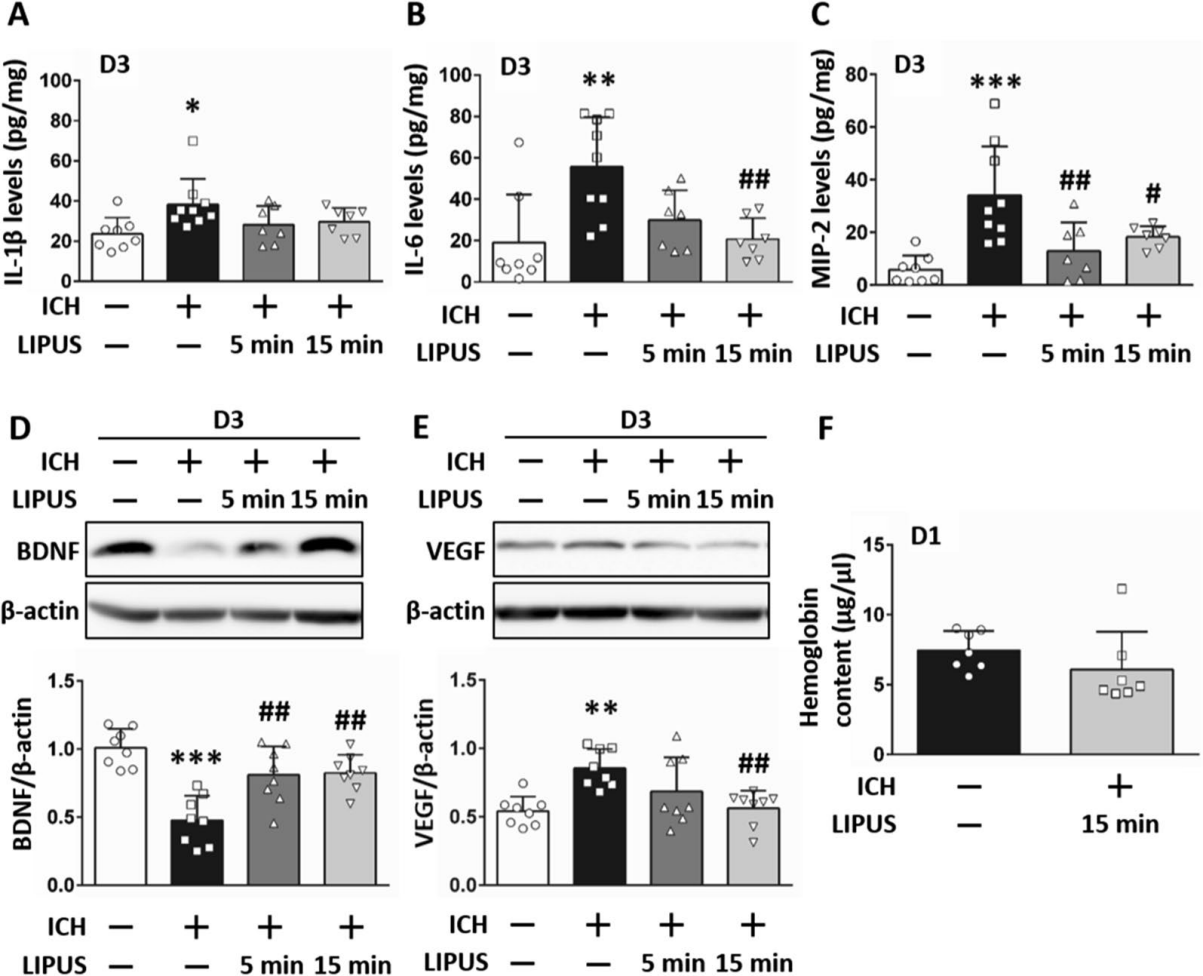
LIPUS treatment reduced inflammatory factor expression after ICH. ELISA analysis indicated that **A** IL-1β, **B** IL-6, and **C** MIP-2 levels were reduced after LIPUS treatment of 5 min and 15 min post-ICH. Western blot analysis also showed that **D** LIPUS treatment significantly mediated ICH-downregulated BDNF expression. **E** VEGF levels were attenuated with 15 min stimulation but not 5 min. However, **F** there was no difference in hemoglobin content at 1 day after LIPUS treatment. *, # denote significant difference from the sham group and ICH group, respectively (*P < 0.05, **, ##*P* < 0.01, ***P < 0.001; n = 7–9)

To evaluate the protective effects and treatment conditions of LIPUS, we measured both inflammatory factors and neurotrophic factors after ICH. After ICH induced the expression of cytokines IL-1β (Fig. 3A), IL-6, and MIP-2 after 3 days compared to sham groups, treatment with LIPUS for 15 min reduced the levels of IL-6 (20.67 ± 10.19 vs 55.73 ± 23.85;* P* = 0.008; Fig. 3B) and MIP-2 (12.90 ± 10.94 at 5 min, 18.30 ± 4.04 at 15 min vs ICH: 34.15 ± 18.65; = 0.004 and* P* = 0.04, respectively; Fig. 3C) expression on D3 post-ICH compared with ICH groups. IL-1β levels were similar between LIPUS 5 min and 15 min treatments and sham groups. BDNF relates to neuron survival after ICH [4] and treatment with LIPUS for 5 min and 15 min significantly corrected ICH-downregulated levels of BDNF expression at 3 days post-injury (0.81 ± 0.21 at 5 min, 0.83 ± 0.13 at 15 min vs ICH: 0.48 ± 0.18;* P* = 0.003 and* P* = 0.002, respectively; Fig. 3D). Another factor, VEGF, has been shown to be associated with hemorrhage production [35] and the results showed that ICH-induced VEGF expression was attenuated after 15-min LIPUS treatment but not after 5-min treatment (0.56 ± 0.13 vs 0.86 ± 0.14;* P* = 0.008; Fig. 3E). However, there was no difference in hemoglobin content between the ICH group and the ICH + LIPUS group one day after ICH (6.08 ± 2.71 vs 7.46 ± 1.39; *P* = 0.255; Fig. 3F). These data indicate that treatment with LIPUS decreased the expression of harmful factors and induced that of protective factors. Further, there was no difference in inflammatory factors and neurotrophic factors between the 5-min treatment and 15-min treatment. Treatment with LIPUS at 15 min was relatively efficient in IL-6 levels and VEGF expression. Thus, we used a 15-min LIPUS treatment for the following experiments.

### Treatment with LIPUS decreased neurological deficits and tissue damage after ICH

**Fig. 4:**
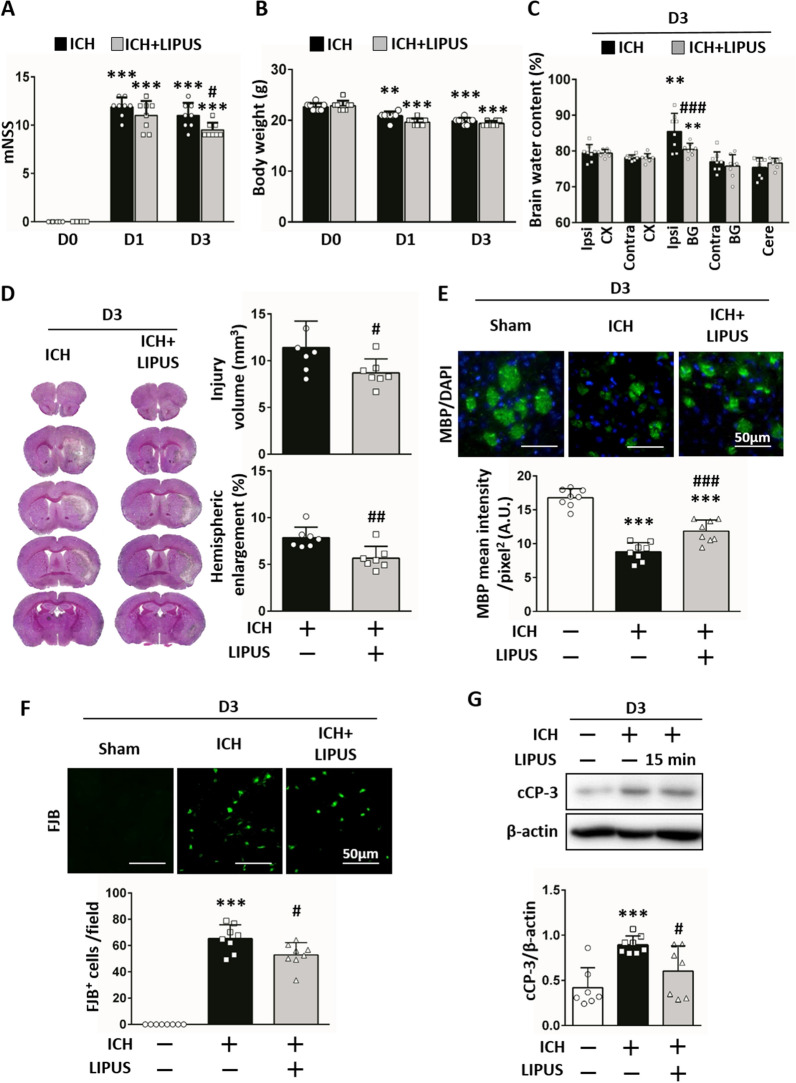
LIPUS treatment decreased neurological deficits and brain damage after ICH. **A** LIPUS significantly improved the mNSS on 3 days post-ICH (***P < 0.001 vs D0, #*P* < 0.05 vs ICH; n = 8). **B** There were no significant differences in body weight between the ICH and ICH + LIPUS groups in the 3 days after ICH (**P < 0.01, ***P < 0.001 versus D0; n = 8). **C** We observed a significant reduction in brain water content in the ipsilateral basal ganglion area of the ICH + LIPUS group compared to the ICH group. (*Ipsi-CX* ipsilateral cortex, *Contra-CX* contralateral cortex, *Ipsi-BG* ipsilateral basal ganglia, *Contra-BG* contralateral basal ganglia, *Cere* cerebellum; **P < 0.01 vs Contra-CX; ###*P* < 0.001 vs ICH at Ipsi-BG; n = 8). Results of histological staining analysis demonstrated that mice in the ICH + LIPUS group exhibited significantly (**D**) lower injury volume and hemispheric enlargement (#*P* < 0.05, ##*P* < 0.01 vs ICH; n = 8), **E** higher myelin basic protein expression (MBP [green], DAPI [blue]; ***P < 0.001 vs sham, ###*P* < 0.001 vs ICH; n = 8), and **F** lower FJB-positive cell numbers than the ICH group (***P < 0.001 vs sham; #*P* < 0.05, ##*P* < 0.01 vs ICH; n = 8). In addition, **G** ICH-induced levels of cleaved caspase-3 expression on D3 were reduced after LIPUS treatment (***P < 0.001 vs sham; #*P* < 0.05, vs ICH; n = 7–8)

Firstly, mNSS was adopted to assess the effects of LIPUS on neurological deficits; the levels of mNSS were elevated dramatically after ICH but were significantly reduced after LIPUS treatment 3 days after injury (9.50 ± 0.76 vs 11.00 ± 1.31, *P* = *0.049*; Fig. 4A). However, there were similar body weight measurements between groups before and after ICH (Fig. 4B). Brain edema is another essential factor to assess ICH outcomes and the results found that brain water content levels were higher at the ipsilateral basal ganglion 3 days post-ICH but were reduced after LIPUS treatment 3 days after ICH (80.45% ± 1.70 vs 85.46% ± 5.04, *P* < 0.001; Fig. 4C). LIPUS treatment also reduced injury volumes (8.71mm^3^ ± 1.48 vs 11.41mm^3^ ± 2.84; *P* = 0.046) and ratios of hemispheric enlargement (5.67% ± 1.26 vs 7.86% ± 1.22; *P* = 0.005) 3 days post-ICH (Fig. 4D). The numbers of neurons and levels of myelin are involved in neurological deficits [13] and tissue damage. Compared with the sham group, ICH significantly reduced MBP expression (*P* < 0.001) and increased the numbers of FJB-positive cells 3 days after ICH (*P* < 0.001); however, LIPUS treatment significantly attenuated the loss of MBP protein expression (11.86 ± 1.63 vs 8.79 ± 1.35, *P* < 0.001; Fig. 4E) and FJB-positive cell numbers (52.88 ± 9.33 vs 65.32 ± 10.46,* P* = 0.017; Fig. 4F) 3 days post-ICH. In addition, ICH-induced levels of cleaved caspase-3 (a cell death marker) expression on D3 were reduced after LIPUS treatment (0.60 ± 0.28 vs 0.89 ± 0.10,* P* = 0.042; Fig. 4G) after ICH. These data suggest that LIPUS treatment after ICH is sufficient to improve acute ICH outcomes. Next, this study sought to identify the possible mechanism by which LIPUS improves ICH outcomes.

### LIPUS treatment decreased glial cell activation after ICH

**Fig. 5:**
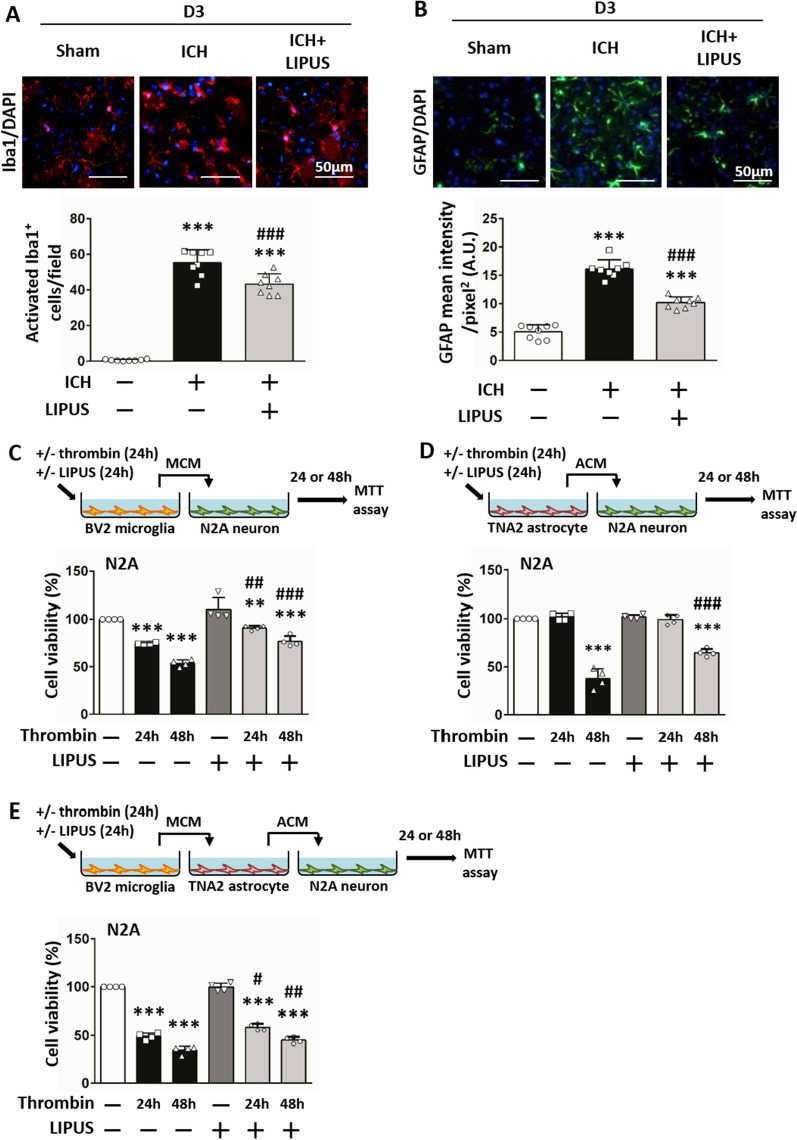
LIPUS treatment decreased glial cell activation after ICH. **A** Microglia (Iba1, red) and **B** astrocytes (GFAP, green) in the peri-hematomal area are shown. LIPUS treatment decreased glial cell expression at 3 days after ICH (n = 8). **C** Microglia-conditioned media (MCM) and **D** astrocyte-conditioned media (ACM) induced by thrombin for 24 h or 48 h caused neuron death. LIPUS treatment prevented MCM- or ACM-caused neurotoxicity. **E** ACM collected from astrocytes activated by thrombin-induced MCM had neurotoxic effects on neuron cultures. LIPUS treatment neutralized the inflammatory activity of thrombin-induced MCM and reduced the cytotoxic effects of ACM. ∗ , #, denote significant difference from the control group and thrombin group, respectively (#*P* < 0.05, **, ##*P* < 0.01, ***, ###*P* < 0.001; n = 4)

Activation of the microglia [8] and astrocytes [16] of glial cells is implicated in post-ICH brain damage. We used immunostaining to investigate the effects of LIPUS on microglial and astrocyte activation. The number of microglia and astrocytes had increased at 3 days after ICH and LIPUS treatment reduced the number of microglia (43.25 ± 5.75 vs 55.29 ± 7.27; *P* < *0*.001; Fig. 5A) and astrocytes (10.20 ± 1.00 vs 16.10 ± 1.63; *P* < 0.001; Fig. 5B). Next, the microglia-conditioned media (MCM) and astrocyte-conditioned media (ACM) experiments were conducted to investigate whether LIPUS treatment reversed microglia- or astrocyte-induced neurotoxicity. Thrombin-induced MCM caused significant neuron death (Fig. 5C). Thrombin-induced ACM also showed neurotoxic activity (Fig. 5D). However, LIPUS treatment prevented MCM- (90.74% ± 2.33 vs 74.53% ± 0.94 at 24 h; 76.90% ± 5.61 vs 53.67% ± 3.79 at 48 h;* P* = 0.003 and *P* < 0.001, respectively; Fig. 5C) and ACM-caused (64.74% ± 3.96 vs 37.84% ± 10.18; *P* < 0.001; Fig. 5D) neurotoxicity after thrombin induction. As microglia increased sooner than astrocytes, we further investigated whether astrocyte-induced neurotoxicity was triggered after microglial activation. Induction with ACM collected from astrocytes that were activated by thrombin-induced MCM was neurotoxic to neuron cultures (*P* < 0.001; Fig. 5E). However, LIPUS treatment neutralized the inflammatory activity of thrombin-induced MCM and, thus, reduced the cytotoxic effects of ACM collected from astrocytes activated by thrombin-induced MCM (58.49% ± 3.53 vs 49.09% ± 3.49 at 24 h,* P* = 0.016;45.24% ± 3.31 vs 34.60% ± 4.13 at 48 h,* P* = 0.005; Fig. 5E). The results suggest that the cytotoxicity of activated microglia can affect neurons directly or indirectly by reactive astrocytes.

### LIPUS treatment controlled the PAR4-PI3K/Akt-NF-κB signaling pathway after microglial activation

**Fig. 6:**
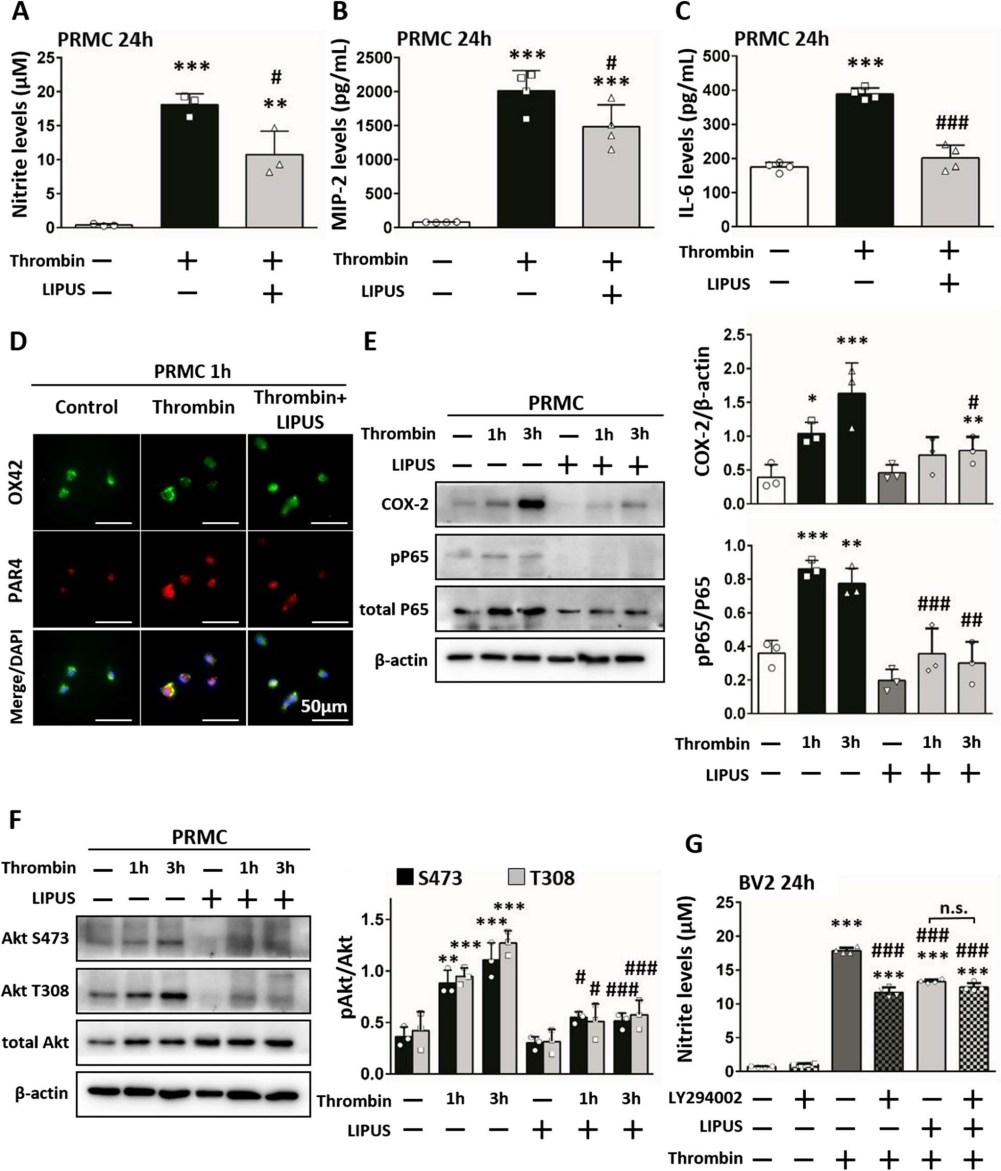
LIPUS treatment controlled the PAR4-PI3K/Akt-NF-κB signaling pathway after glial cells activation. **A** Nitrite, **B** MIP-2 and **C** IL-6 levels in the primary microglia culture (PRMC) were increased by thrombin and significantly decreased microglia activation by LIPUS treatment. **D** Immunofluorescence staining was used to morphologically analyze microglia. OX42 (microglia) is shown in green, and PAR4 (hallmarks of inflammation) is shown in red. Yellow labeling represents co-localization. LIPUS treatment reduced the intensity of PAR4 staining induced by thrombin. DAPI (blue) staining was used to show all nuclei. The scale bar is 50 μm. **E** Representative immunoblots and bar graphs show that LIPUS treatment reduced COX-2 levels and P65 phosphorylation levels and **F** Akt phosphorylation at both S473 and T308 sites induced by thrombin at 1 h and/or 3 h. **G** The PI3K inhibitor LY294002 significantly reduced thrombin-induced nitrite production. The results of the thrombin + LIPUS + LY294002 group were similar to those of the thrombin + LIPUS group (*P* = 0.262). The results suggest that LIPUS treatment ameliorates microglia activation by limiting the PAR4-PI3K/Akt-NF-κB signaling pathway. ∗ , #, denote significant difference from the control group and thrombin group, respectively (*, #*P* < 0.05, **, ##*P* < 0.01, ***, ###*P* < 0.001, n.s. = *P* > 0.05; n = 3–4)

We examined cytokine expression in primary cell cultures to investigate whether LIPUS treatment reduced microglial activation and its potential signaling pathway. LIPUS treatment decreased thrombin-induced nitrite levels (10.73 μM ± 3.46 vs 18.11 μM ± 1.60; *P* = 0.019; Fig. 6A) and MIP-2 (1483.43 pg/mL ± 321.83 vs 2015.47 pg/mL ± 296.52; *P* = 0.046; Fig. 6B) and IL-6 (201.44 pg/mL ± 36.92 vs 388.65 pg/mL ± 17.57; *P* < 0.001; Fig. 6C) levels in primary microglia. PAR4 has been shown to be involved in thrombin-induced microglial activation [9]. We used double immunofluorescence to further analyze the morphology of microglia. Our results showed that PAR4 staining intensity was reduced after LIPUS treatment (Fig. 6D) and thrombin-induced COX-2 (0.79 ± 0.20 vs 1.63 ± 0.45 at 3 h;* P* = 0.024; Fig. 6E) and p65 phosphorylation levels (0.36 ± 0.15 vs 0.86 ± 0.05 at 1 h, *P* < 0.001; 0.30 ± 0.13 vs 0.77 ± 0.09 at 3 h, *P* < 0.001; Fig. 6E) decreased after LIPUS treatment. PI3K/Akt signaling has been reported to induce an inflammatory response [12]. We found that thrombin-induced Akt phosphorylation at both S473 (0.55 ± 0.05 vs 0.88 ± 0.13 at 1 h,* P* = 0.032; 0.51 ± 0.08 vs 1.11 ± 0.17 at 3 h, *P* < 0.001; Fig. 6F) and T308 (0.51 ± 0.17 vs 0.95 ± 0.08 at 1 h,* P* = 0.033; 0.57 ± 0.14 vs 1.27 ± 0.12 at 3 h, *P* < 0.001; Fig. 6F) sites in primary microglia was reversed after LIPUS treatment. Next, the PI3K inhibitor LY294002 was given to confirm the effects of PI3K/Akt signaling after LIPUS treatment. Thrombin-induced nitrite production in BV2 microglia was reduced after inhibition with LY294002 (11.70 μM ± 0.74 vs 17.86 μM ± 0.46; *P* < 0.001; Fig. 6G) and nitrite production was similar between the thrombin + LIPUS group and the thrombin + LIPUS + LY294002 group (12.48 μM ± 0.61 vs 13.31 μM ± 0.27; *P* = 0.262; Fig. 6G). The results suggest that PI3K/Akt signaling is activated by thrombin and the anti-inflammatory effects of LIPUS result from inhibiting the PI3K/Akt signaling pathway to ameliorate microglial activation.

### LIPUS treatment decreased cytotoxic effects after astrocyte activation

**Fig. 7:**
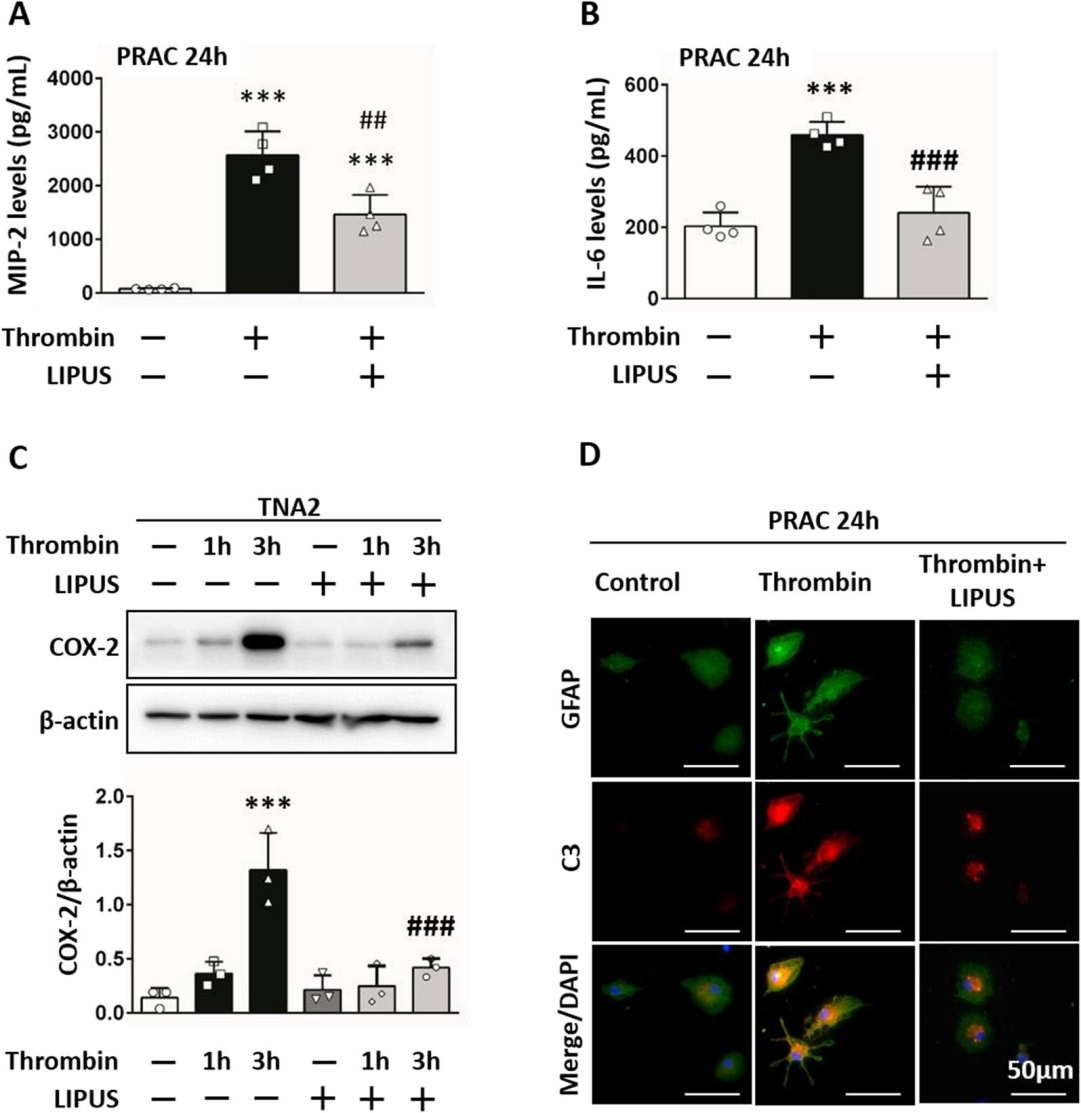
LIPUS treatment decreased cytotoxic effects after astrocyte activation. **A** MIP-2, **B** IL-6 and **C** COX-2 expression in the primary astrocyte culture (PRAC) were increased by thrombin and significantly decreased after LIPUS treatment. **D** Immunofluorescence staining was used to morphologically analyze astrocytes. GFAP (astrocytes) is shown in green and C3 (hallmarks of cytotoxic) is shown in red. Yellow labeling represents co-localization. LIPUS treatment reduced the expression of C3 staining induced by thrombin. DAPI (blue) staining was used to show all nuclei. The scale bar is 50 μm. ∗ , #, denote significant difference from the control group and thrombin group, respectively (## = *P* < 0.01, ***, ### = *P* < 0.001; n = 4)

The expression of cytokines MIP-2 and IL-6 induced by thrombin were decreased after LIPUS treatment (MIP-2: 1460.89 pg/mL ± 364.75 vs 2567.41 pg/mL ± 443.66,* P* = 0.003, Fig. 7A; IL-6: 240.55 pg/mL ± 73.60 vs 459.29 pg/mL ± 36.76, *P* < 0.001, Fig. 7B). The thrombin-induced levels of COX-2 were also decreased after LIPUS treatment (0.59 ± 0.08 vs 1.32 ± 0.34; *P* < 0.001; Fig. 7C). Meanwhile, the cytotoxic marker C3 was upregulated in primary astrocytes after thrombin induction and was reduced after LIPUS treatment (Fig. 7D). These data suggest that thrombin directly induces astrocyte activation to exert cytotoxicity.

### Delayed LIPUS treatment still reduces brain damage and glial activation after ICH

**Fig. 8:**
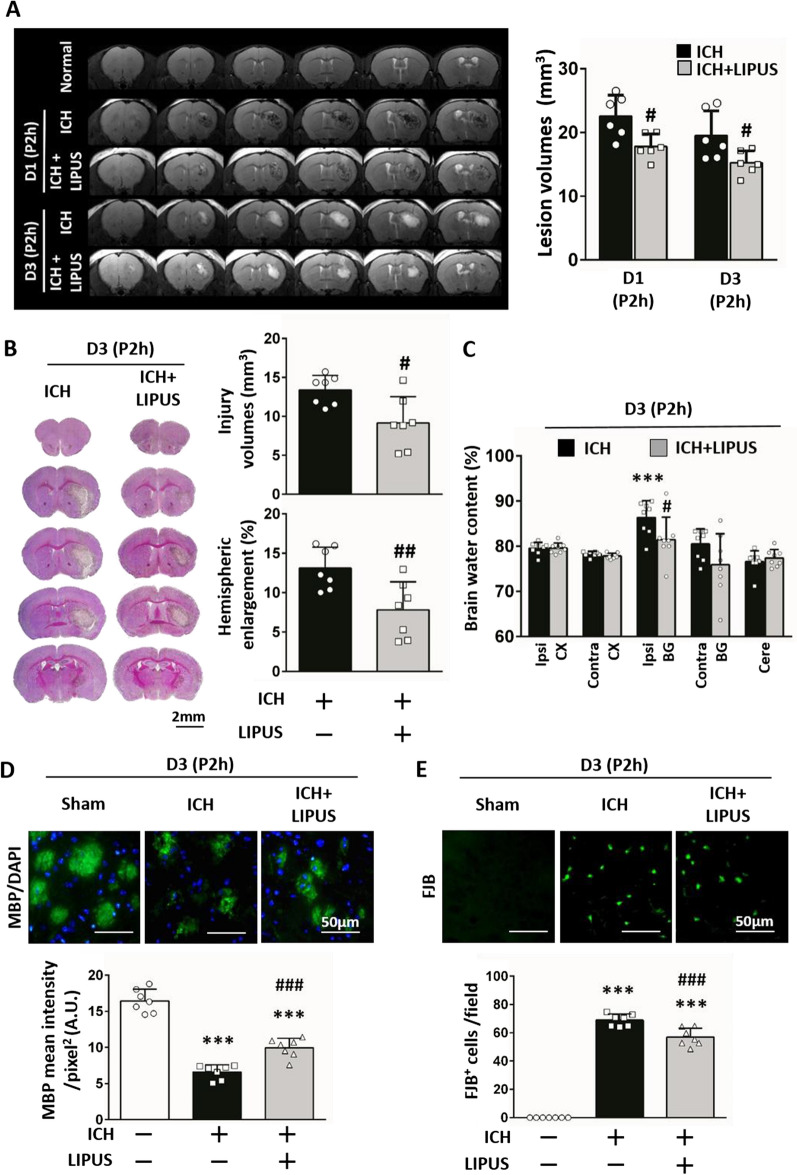
Delayed LIPUS intervention still reduced brain damage after ICH. **A** Representative MRI T2-weighted images (T2WIs) at 1 day and 3 days post-ICH. LIPUS treatment performed two hours (P2h) after ICH. The contour of lesion volumes was defined by the contrast provided by T2WIs of the brain. Quantification revealed that lesion volumes were significantly reduced after delayed LIPUS intervention. **B** Cresyl violet staining showed injury volumes and hemispheric enlargement were significantly decreased after delayed LIPUS treatment (scale bar: 2 mm). **C** Results of brain water content demonstrated that the ICH + LIPUS group exhibited significantly less brain edema than the ICH group in the ipsilateral basal ganglia area at D3. (Ipsi-CX: ipsilateral cortex, Contra-CX: contralateral cortex, Ipsi-BG: ipsilateral basal ganglia, Contra-BG: contralateral basal ganglia, Cere: cerebellum) **D** Representative MBP-stained and **E** FJB-stained sections of a sham, an ICH, and a delayed LIPUS-treated group at 3 days post-ICH. ICH-induced myelin loss and neurodegeneration were significantly ameliorated after delayed LIPUS treatment (scale bar: 50 mm). ∗ , #, denote significant difference from the sham group and ICH group, respectively (#*P* < 0.05, ##*P* < 0.01, ***, ###*P* < 0.001; n = 6–8)

To evaluate the potential clinical utility of LIPUS treatment, we conducted further research to determine whether the protective effects persist with a delayed intervention of 2 h after the onset of ICH. We evaluated the lesion size using MRI T2WIs and found that the lesion volumes were significantly reduced after LIPUS treatment at both 1 and 3 days following ICH (17.81mm^3^ ± 1.96 vs 22.56 mm^3^ ± 3.3 at D1,* P* = 0.013; 15.24mm^3^ ± 1.88 vs 19.55 mm^3^ ± 3.85 at D3,* P* = 0.034; Fig. 8A). The results also showed that LIPUS treatment significantly decreased injury volumes (9.16 mm3 ± 3.68 vs 13.38 mm3 ± 2.00; *P* = 0.013), hemispheric enlargement (7.80% ± 3.44 vs 13.10% ± 2.88; *P* = 0.008; Fig. 8B), brain water content levels in the ipsilateral basal ganglia area (81.46% ± 5.01 vs 86.34% ± 3.75; *P* = 0.044; Fig. 8C), myelin loss (9.95 ± 1.32 vs 6.61 ± 0.99; *P* < 0.001; Fig. 8D) and neurodegeneration (56.92 ± 6.27 vs 68.95 ± 4.32; *P* < 0.001; Fig. 8E).

**Fig. 9:**
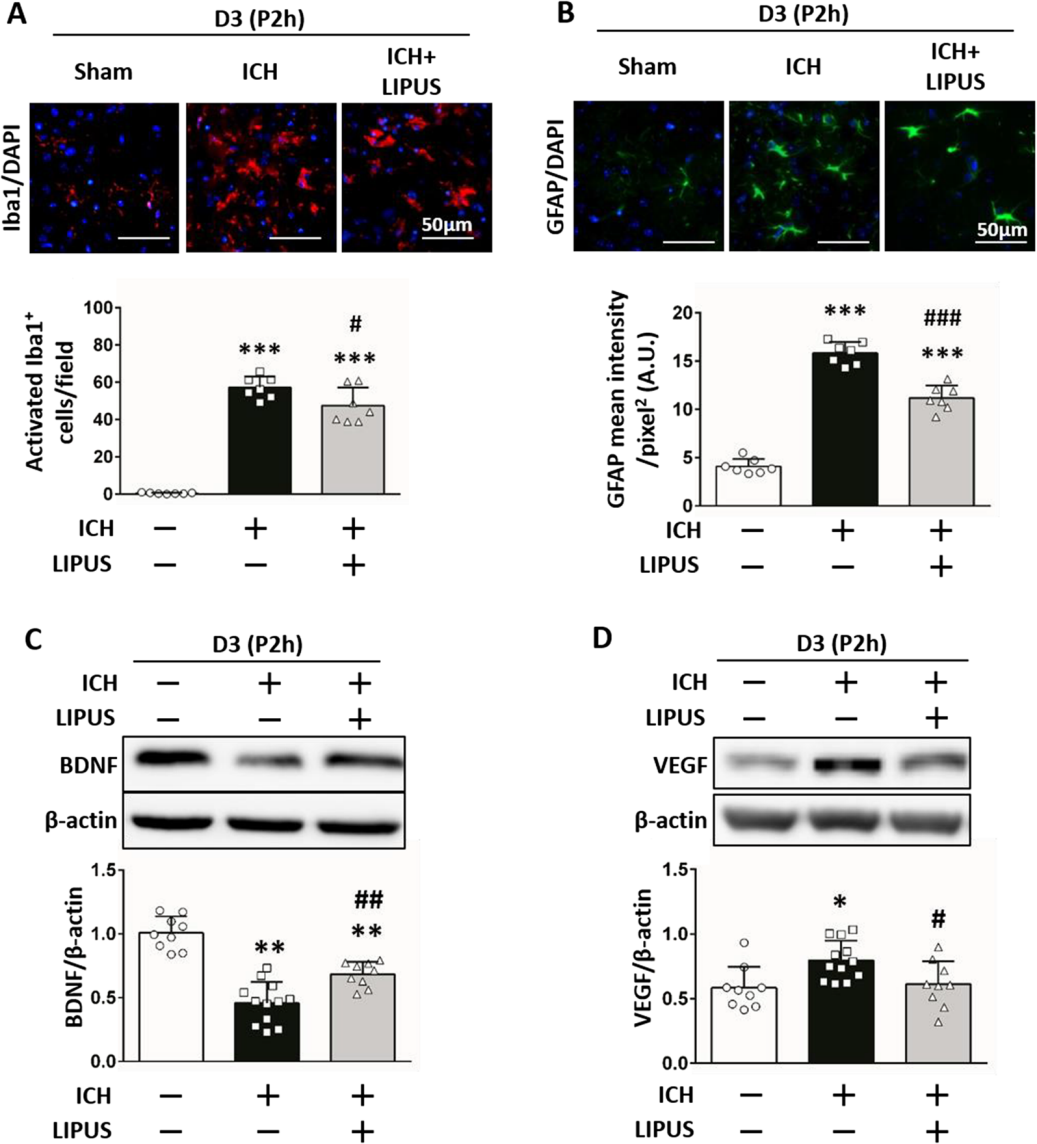
Amelioration of glial activation and neurotrophic levels by delayed LIPUS intervention following ICH **A** Iba1-positive numbers (microglia, red), and **B** GFAP intensity (astrocyte, green) represented activation of microglia and astrocytes, respectively. Quantification analysis shows that delayed LIPUS treatment also significantly ameliorated glial cell activation at 3 days post-ICH. (Nuclei were stained with DAPI [blue]; scale bar: 50 mm). **C** Representative ICH-downregulated BDNF expression, and **D** ICH-upregulated VEGF expression were significantly reversed after delayed LIPUS treatment. ∗ , #, denote significant difference from the sham group and ICH group, respectively (*, #*P* < 0.05, **, ##*P* < 0.01, ***, ###*P* < 0.001; n = 6 – 8)

Glia-relative markers of Iba1 (47.46 ± 9.69 vs 57.19 ± 5.87; *P* = 0.037; Fig. 9A) and GFAP (11.18 ± 1.30 vs 15.82 ± 1.15; *P* < 0.001; Fig. 9B) were also ameliorated after LIPUS treatment. ICH-downregulated levels of BDNF (0.68 ± 0.10 vs 0.46 ± 0.16;* P* = 0.002; Fig. 9C) and ICH-upregulated levels of VEGF (0.61 ± 0.18 vs 0.80 ± 0.15; *P* = 0.043; Fig. 9D) were also significantly improved. These findings suggest that LIPUS treatment may be a viable clinical option for ICH, even by LIPUS treatment despite the 2-h delay.

## Discussion

This study demonstrated that LIPUS ameliorated ICH-induced neurological deficits and reduced brain damage through tissue loss, edema, and neurodegeneration. The protective effects of LIPUS in ICH were enacted by decreasing the inflammatory response of microglial activation and levels of reactive astrocytes after ICH. Mechanistically, LIPUS inhibited PI3K/Akt-NF-κB signaling to reduce MIP-2 and IL-6 expression and attenuate microglial activation-caused neuronal damage in vitro. In addition, LIPUS reduced microglial activation-induced neurotoxicity in reactive astrocytes. Moreover, LIPUS reduced the reactive astrocyte levels directly. We further found that LIPUS has significant protective effects in ICH in a more clinically relevant 2-h-delayed treatment. Our findings indicate that LIPUS is a potential non-invasive therapy for ICH.

Hematoma expansion and brain edema formation are two important signs to predict ICH outcomes [36], and our results showed that LIPUS treatment has the advantage of reducing brain water content levels after ICH and did not produce further hemoglobin content expression post-injury. In addition, the critical temperature needs to be carefully controlled in patients suffering from ICH [37], however, previous study indicates that temperature fluctuations of only 0.5 °C after 10 min of LIPUS exposure, so the thermal effect of LIPUS treatment could be ignored [38]. In clinical, using surgery to remove clots limits to favorable locations such as the lobar, cerebellar, external capsule, and non-dominant hemisphere after ICH, In the present study, when the hemorrhage is located in the basal ganglia, an unfavorable location for surgery, LIPUS treatment still has the advantage to improve outcomes after ICH production. Overall, LIPUS has the therapeutic potential to treat ICH disease clinically.

Inflammation is an important issue in ICH [2, 7] and microglia and astrocytes are two resident immune cell types that modulate the brain’s inflammatory response after ICH [2]. We confirmed that both microglia and astrocytes were upregulated after ICH-induced injury. In this study, microglia expression increased at 3 h after ICH, similar to previous studies showing a 20% upregulation of microglia 6 h after collagenase-induced rat ICH [39] and increased biomarkers TNF-α and IL-6 from activated microglia 3 h after ICH [40]. Astrocyte levels were upregulated 3 days post-injury, aligning with increased GFAP levels 3 days after ICH [41]. Although another study showed that astrocyte numbers increased acutely from 3 to 12 h after ICH [16], the severity of their ICH model (collagenase concentration: 0.025U/0.5μL) was also milder than that in this study (0.0375U/1μL). This suggests that astrocyte activation might differ by ICH severity. Microglia could be activated earlier than astrocytes in ICH and may represent an upstream factor for astrocyte activation [19].

Previous study showed that inhibiting microglial activity ameliorated ICH outcomes [8]. Our results found that ICH-induced inflammatory cytokines and microglia levels were reduced after LIPUS treatment. Similarly, LIPUS attenuated thrombin-induced MCM causing neuronal death. These results confirm the anti-inflammatory effects of LIPUS in ICH. We found that thrombin-induced PI3K/Akt-NF-κB signaling was reduced after LIPUS treatment in microglial culture. However, our previous study showed that thrombin could induce phosphorylation of JNK, P38, and ERK in MAPK pathways to activate P65 phosphorylation in microglia [33]. In visceral preadipocytes, ultrasound contributes to P38 and ERK phosphorylation [42]. Thus, further investigation to explore the relationship between MAPK signaling and LIPUS treatment in microglia after thrombin induction is needed. However, the anti-inflammatory effects of LIPUS in microglia after thrombin induction were absent when a PI3K/Akt inhibitor was administered before LIPUS intervention. Logically, LIPUS dominantly inhibits PI3K/Akt-NF-κB signaling, reducing microglial activation and its neurotoxic effects. Moreover, recent data showed upregulated astrocyte activation (C3-positive astrocytes) after treatment with MCM [43] and our results showed that ACM harvested from thrombin-induced MCM to cause neuronal death was reduced after LIPUS treatment. Thus, the neuroprotective effects of LIPUS may either inhibit microglia-induced neurotoxicity or reduce microglia-induced astrocytic neurotoxicity.

In addition to microglia, the inhibition of astrocyte activity also improved ICH outcomes [16]. LIPUS reduced astrocyte intensity after ICH. Recent studies showed that knocking out PAR-1 accelerated myelin development in the brain [44]. An astrocyte that lacks PAR-1 expression induced levels of proteolipid protein and MBP expression in oligodendrocytes [44]. We found decreased neurotoxic effects and cytokine expression in thrombin-induced ACM after LIPUS treatment. One possible mechanism for thrombin-induced primary astrocyte activation to increase cytokine expression is through the PAR-1/SphK/S1P axis [45]. The direct regulating mechanism of LIPUS to reduce thrombin-induced astrocyte activation in ICH must be explored in the future.

Previous studies suggested that LIPUS induced BDNF expression in the brain post-TBI [23]. Direct LIPUS treatment induced BDNF expression and suppressed VEGF expression in microglia during hypoxia/reperfusion [46]. BDNF expression is important for neuron survival in the brain [47] but is decreased after ICH [4]. Thus, we speculate that LIPUS treatment may induce BDNF expression or decrease VEGF expression in microglia after ICH. In contrast, another study indicated that N9 microglial activation after LPS induction can induce BDNF expression [48]. Different cell lines may display different BDNF production in microglia, although we did not assess BDNF levels in the culture medium from thrombin-induced primary microglia or the BV2 microglial cell line. Compared with thrombin induction signaling, LPS also induces the PI3K/Akt pathway through the TLR4 receptor in microglial culture [49]. Another possibility is that different inductions may cause different BDNF-producing events in microglia. Further studies are needed to clarify this issue. VEGF has many roles in the brain, including angiogenesis [50], hemorrhage formation [51], and edema formation [52]. LIPUS treatment reduced VEGF expression after ICH and was related to decrease levels of brain water content but did not affect hemoglobin levels after ICH. So, the effects of LIPUS on reducing VEGF expression may be associated with decreasing brain edema levels after ICH.

We performed a 2-h-delayed LIPUS intervention after ICH to mimic clinical usage. From the results of the MRI and histology, we found that LIPUS treatment could alleviate the levels of brain swelling, edema, and degeneration of neurons and myelin despite the 2-h delay. The results also showed significant improvements that expressions of microglia and astrocytes were reduced after the delayed LIPUS treatment. The protein expression of BDNF and VEGF also showed consistent results.

There are some limitations of this study as described, at first, we only observed the reduced neurodegenerative levels after ICH after LIPUS treatment. Our previous study reported the neuroprotective effects of LIPUS through anti-apoptosis in TBI [30]. Previous studies indicated that LIPUS regulated the differentiation levels of neural stem cells toward more NeuN-positive but not GFAP-positive cells [53], which suggests a neurogenesis role. Thus, whether LIPUS could directly regulate neuron survival or neurogenesis after ICH-induced neuronal loss remains to be investigated. Another concern is that although LIPUS improved neurological deficits at day 3 after ICH, the long-term improvement of neurological deficits after LIPUS treatment in ICH is yet unknown. However, collagenase-induced ICH model cannot simply represent clinical ICH disease, the protective effects of LIPUS in ICH needs to apply in different ICH models such as the autologous blood injection ICH model [54]. And the time window and the treatment days of LIPUS after ICH need to be extent.

## Conclusions

**Fig. 10:**
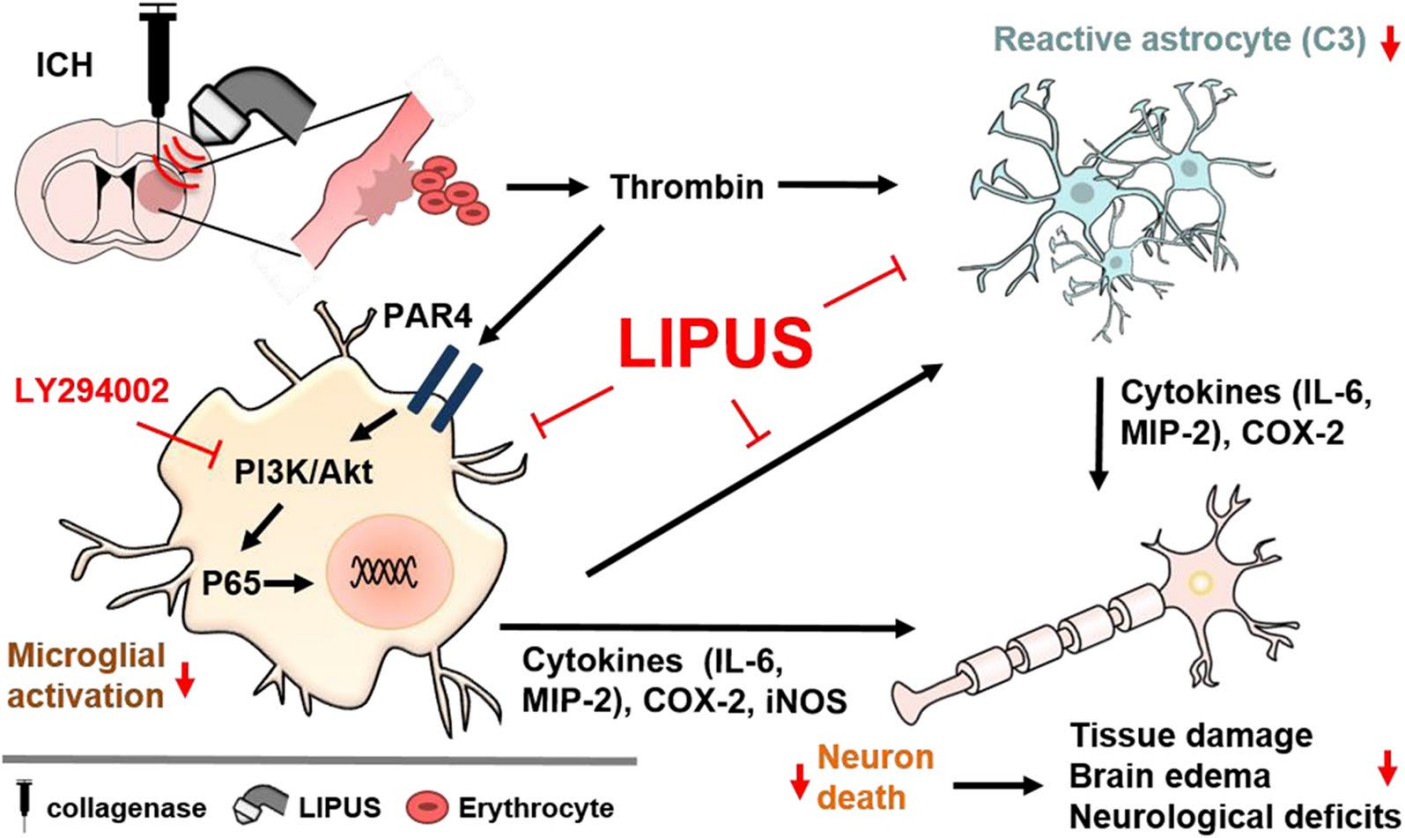
Schematic diagram of LIPUS-improved ICH outcomes and microglial and astrocyte expression. Following ICH, erythrocytes accumulate around damaged cerebral vessels, and thrombin not only aids in blood coagulation but also activates microglia and astrocytes. LIPUS treatment regulates the PI3K/Akt-NF-κB signaling pathways, reducing microglia-induced neurotoxicity and inhibiting astrocytic neurotoxicity, which contributes to the amelioration of ICH outcomes. LIPUS intervention after ICH also promotes anti-inflammatory effects and decreases apoptosis

As summarized in Fig. 10, LIPUS treatment ameliorates glia-mediated inflammation and neuronal damage by reducing PI3K/Akt-NF-κB signaling to inhibit microglia-induced neurotoxicity. Meanwhile, suppressing astrocytic neurotoxicity directly or indirectly also contributes to the effectiveness of LIPUS treatment. Our findings suggest that LIPUS treatment presents a non-invasive potential ICH management strategy.

### Supplementary Information

**Additional file 1: Figure S1**. Schematic diagram of quantification of FJB and immunostaining. Three consecutive coronal sections of the core hemorrhagic region in the brain atlas (AP, + 0.24 mm from the bregma) were analyzed for each mouse. The three black boxes around the hemorrhage core indicate the location of representative images.

## Data Availability

The data used and/or analyzed during the current study are available from the corresponding author on reasonable request.

## Abbreviations

ICH: Intracerebral hemorrhage
LIPUS: Low-intensity pulsed ultrasound
IL: Interleukin
PAR4: Protease-activated receptor 4
MIP-2: Macrophage inflammatory protein-2
PI3K: Phosphoinositide 3-kinase
cCP-3: Cleaved caspase-3
COX-2: Cyclooxygenase-2
NO: Nitrite oxide
iNOS: Inducible nitric oxide synthase
BDNF: Brain-derived neurotrophic factor
VEGF: Vascular endothelial growth factor
MCM: Microglia-conditioned media
ACM: Astrocyte-conditioned media
MBP: Myelin basic protein
