# Supplementary material for: Low-intensity pulsed ultrasound ameliorates glia-mediated inflammation and neuronal damage in experimental intracerebral hemorrhage conditions
Source: J Transl Med. 2023 Aug 24;21:565. doi: 10.1186/s12967-023-04377-z (PMC10464049; doi:10.1186/s12967-023-04377-z)
Supplement: Supplementary file 1 — Additional file 1: Figure S1. Schematic diagram of quantification of FJB and immunostaining. Three consecutive coronal sections of the core hemorrhagic region in the brain atlas (AP, + 0.24 mm from the bregma) were analyzed for each mouse. The three black boxes around the hemorrhage core indicate the location of representative images. [file 12967_2023_4377_MOESM1_ESM.docx]

**Additional File 1**

**Additional Figure.**


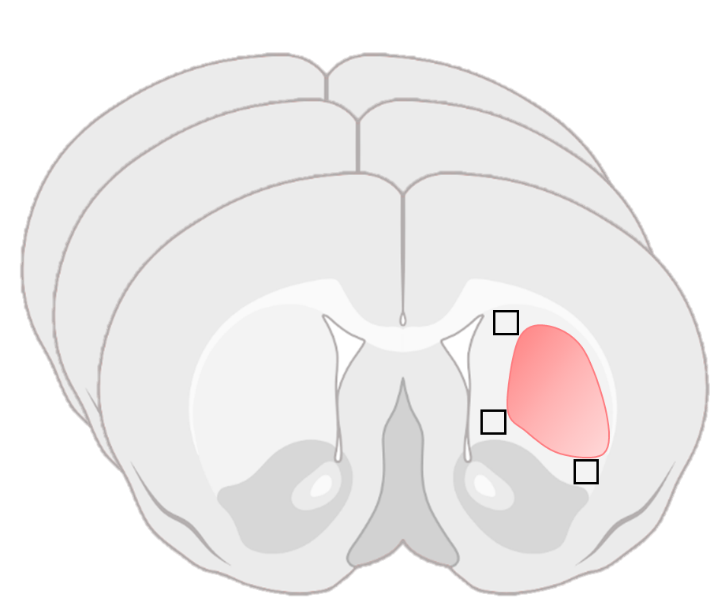


**Figure S1. Schematic diagram of quantification of FJB and immunostaining.** Three consecutive coronal sections of the core hemorrhagic region in the brain atlas (AP, +0.24 mm from the bregma) were analyzed for each mouse. The three black boxes around the hemorrhage core indicate the location of representative images.
